# Synthesis of a Low-Cost Thiophene-Indoloquinoxaline Polymer Donor and Its Application to Polymer Solar Cells

**DOI:** 10.3390/polym14081554

**Published:** 2022-04-11

**Authors:** Yiping Guo, Zeyang Li, Mengzhen Sha, Ping Deng, Xinyu Lin, Jun Li, Liang Zhang, Hang Yin, Hongbing Zhan

**Affiliations:** 1 College of Materials Science and Engineering, Fuzhou University, Fuzhou 350108, China; gyp010601@163.com (Y.G.); n191820032@fzu.edu.cn (Z.L.); lxy153694@163.com (X.L.); lj03040010@163.com (J.L.); 18379452130@139.com (L.Z.); hbzhan@fzu.edu.cn (H.Z.); 2 State Key Laboratory of Crystal Materials, School of Physics, Shandong University, Jinan 250100, China; 202112136@mail.sdu.edu.cn; 3 Key Laboratory of Eco-materials Advanced Technology Fuzhou University, Fuzhou 350108, China

**Keywords:** indoloquinoxaline, low-cost polymer donor, wide-bandgap polymer, polymer solar cells

## Abstract

A simple wide-bandgap conjugated polymer based on indoloquinoxaline unit (**PIQ**) has been newly designed and synthesized via cheap and commercially available starting materials. The basic physicochemical properties of the **PIQ** have been investigated. **PIQ** possesses a broad and strong absorption band in the wavelength range of 400~660 nm with a bandgap of 1.80 eV and lower-lying highest occupied molecular orbital energy level of −5.58 eV. Polymer solar cells based on **PIQ** and popular acceptor Y6 blend display a preliminarily optimized power conversion efficiency of 6.4%. The results demonstrate indoloquinoxaline is a promising building unit for designing polymer donor materials for polymer solar cells.

## 1. Introduction

Polymer solar cells (PSCs) are attractive as a promising new energy device for solar-to-electric conversion [1]. In a typical device, the active-layer blending film consists of a donor material and an acceptor material [2,3]. One of the most successful blends in recent years contains a p-type polymer as the donor and an n-type non-fullerene molecule as the acceptor [4]. Thanks to rational molecular design and device optimization [5], the power conversion efficiencies (PCEs) of PSCs have consistently improved [6]. However, one issue that must be critically considered is the cost of the active-layer materials [7,8]. Because of the complicated molecular structures, tedious multi-step organic synthesis, and laborious purifications, the costs of the efficient active-layer materials reported so far were too high to meet commercial application of PSCs [9]. Therefore, developing low-cost and efficient active-layer materials is one of the key challenges for the application of PSCs [10,11].

**Figure 1:**
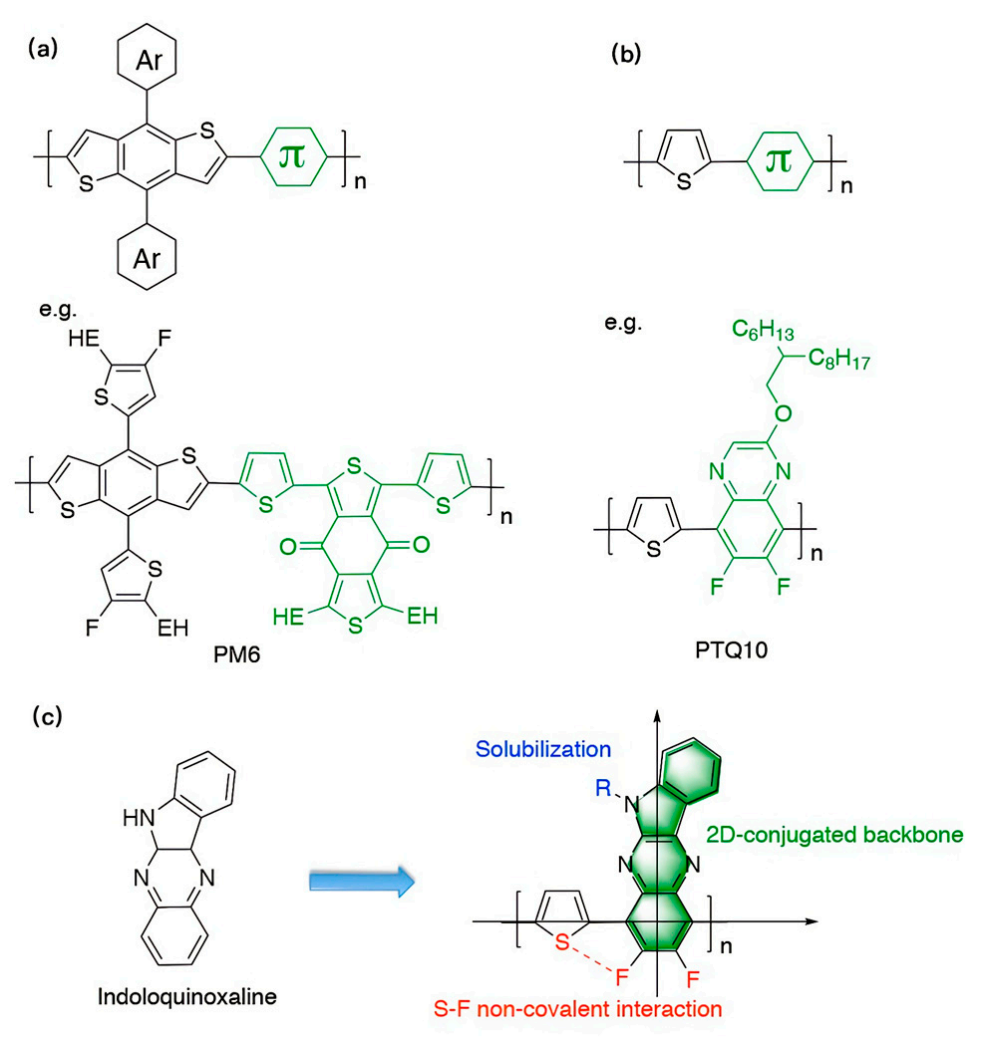
(**a**) BDT-based and (**b**) thiophene-based polymer donors; (**c**) molecular design strategy of the indoloquinoxaline-based polymer.

Recently, a low-cost and high-performance polymer donor, PTQ10 [12], has been demonstrated as a promising polymer donor for commercial application of PSCs. Compared to the classical benzo [1,2-*b*:4,5-*b′*] dithiophene (BDT)-based polymers [13,14,15] (see Figure 1a) synthesized via multi-step synthesis, PTQ10 has a very simple molecular structure (see Figure 1b), and it can be synthesized via simple two-step reactions with cheap raw materials. Low-cost and efficient polymer donors have gained relative less attention in recent years, and only a few polymers with these features have been developed until now [9]. We had reviewed and summarized the representative low-cost and efficient polymer donors (see Table S1 and Figure S7). It was shown that these types of polymers are promising donor materials for high-performance PSCs [16,17,18,19,20,21,22]. Therefore, we expected to develop new low-cost and efficient polymer donors.

Indolo[2,3-*b*] quinoxaline (IQ) is a unique planar built-in donor–acceptor heterocyclic unit that can be considered as the fusion of electron-deficient quinoxaline and electron-rich indole. Some IQ small molecular derivatives have been applied as promising multifunctional anti-Alzheimer agents [23], photosensitizers [24,25,26], hole injection-layer materials [27] and non-fullerene acceptors [28]. In this work, we designed an IQ-based polymeric p-type semiconductor material (**PIQ**) for polymer solar cells. The molecular design strategy is shown in Figure 1c. This polymer contains simple thiophene and difluorine-substituted IQ units with two-dimensional (2D) conjugated backbone. The 2D conjugated structure is favorable for intermolecular carrier transporting [29,30,31]. The fluorination of the IQ unit is to improve molecular planarity via S···F non-covalent interactions and further enhance carrier transporting [32,33,34,35]. The alkyl side chain on the IQ unit is to ensure good solubility.

## 2. Materials and Methods

### 2.1. Materials

**Scheme 1:**
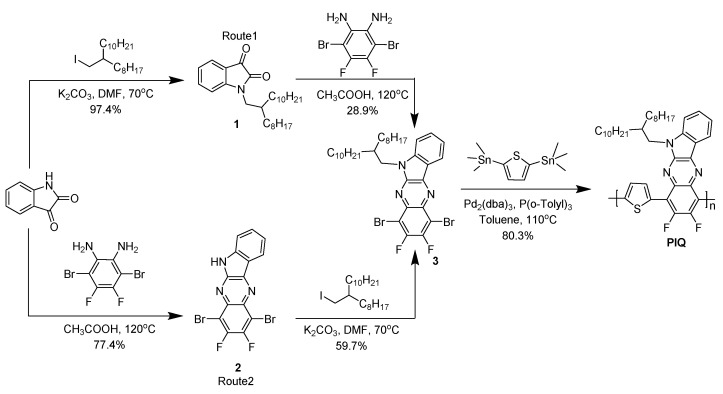
Synthetic routes of the **PIQ**.

9-(Iodomethyl)nonadecane (97%, Lyntech), 3,6-Dibromo-4,5-difluorobenzene-1,2- diamine (98%, Zhengzhou Ruke Biological, Zhengzhou, China), indoline-2,3-dione (97%, Rhawn), and 2,5-bis(trimethylstannyl)thiophene (99%, bidepharm), potassium carbonate (K_2_CO_3_, 98%, Aladdin), *N*,*N*-Dimethylformamide (DMF, AR, 99.5%, Aladdin), toluene (99.5%, Aladdin), acetic acid (CH_3_COOH, 99.7%, Aladdin), Tris(dibenzylideneacetone)dipalladium(0) (Pd_2_(dba)_3_, 97%, Aladdin), Tri(*o*-tolyl)phosphine (P(*o*-tolyl)_3_, 97%, Aladdin), calcium hydride (95%, Aladdin), molecular sieves (3Å, Aladdin), 2,2′-((2Z,2′Z)-((12,13-bis(2-ethylhexyl)-3,9-diundecyl-12,13-dihydro-[1,2,5]thiadiazolo-[3,4-*e*]thieno[2”,3′’:4′,5′]thieno[2′,3′:4,5]pyrrolo[3,2-*g*]thieno[2′,3′:4,5]thieno[3,2-*b*]indole-2,10-diyl)bis(methanylylidene))bis(5,6-difluoro-3-oxo-2,3-dihydro-1*H*-inde (Y6, 98%, Zhengzhou Alfachem Co., Ltd., Zhengzhou, China) were used as received. Toluene was distilled over calcium hydride under an argon atmosphere and was then dried with 3Å molecular sieves. The detailed synthesis routes are shown in Scheme 1.

### 2.2. Synthesis of 1,4-Dibromo-2,3-difluoro-6-(2-octyldodecyl)-6H-indolo[2,3-b]quinoxaline

[Route 1] 1-(2-octyldodecyl) indoline-2,3-dione (**1**) [35] was synthesized according to literature procedures. Quantities of 3,6-dibromo-4,5-difluorobenzene-1,2-diamine (0.66 mmol, 0.2033 g) and 1-(2-octyldodecyl)indoline-2,3-dione (0.54 mmol, 0.2309 g) were added to a Schlenk reaction flask (38 mL) under an argon atmosphere, followed by the addition of deoxygenated acetic acid (3.5 mL), and the reaction was carried out at 120 °C for 16 h. After the reaction was cooled to room temperature, the mixture was poured into cold water (100 mL). It was then extracted with dichloromethane (50 mL × 3). The combined organic layers were washed with water and brine then dried over anhydrous magnesium sulfate. After removing the solvent, the crude product was purified by flash column chromatography (silica gel, dichloromethane: petroleum ether = 1:2, *v*/*v*) to afford the titled compound (0.1083 g, 28.9%) as a yellow solid.

[Route 2] Indoline-2,3-dione (0.66 mmol, 0.0991 g) and 3,6-dibromo-4,5-difluorobenzene-1,2-diamine (0.55 mmol, 0.1694 g) were added to a Schlenk reaction flask (38 mL) under argon atmosphere, followed by the addition of deoxygenated acetic acid (1.8 mL), and the reaction was carried out at 120 °C for 16 h. After the reaction was cooled to room temperature, the mixture was poured into water (100 mL). The precipitate was filtered and then washed with methanol (5 mL × 4) and dried under vacuum to obtain the 1,4-dibromo-2,3-difluoro-6*H*-indolo[2,3-*b*]quinoxaline (0.1759 g, 77.4%) as a yellow solid. Next, 1,4-dibromo-2,3-difluoro-6*H*-indolo[2,3-*b*]- quinoxaline was transferred to a double-necked flask (250 mL) under argon atmosphere. K_2_CO_3_ (0.86 mmol, 0.1189 g), and deoxygenated DMF (2 mL) were added. Subsequently, 9-(iodomethyl)nonadecane (0.65 mmol, 0.2709 g) was added slowly dropwise. The reaction was carried out at 70 °C for 21 h. After the reaction was cooled to room temperature, the product was poured into water (100 mL). It was then extracted with dichloromethane (50 mL × 3). The combined organic layers were washed with water and brine and dried over anhydrous magnesium sulfate. After removing the solvent, the crude product was purified by flash column chromatography (silica gel, dichloromethane: petroleum ether = 1:4, *v*/*v*) to afford the titled compound (0.1779 g, 59.7%) as a yellow solid.

**^1^H NMR** (400 MHz, CDCl_3_, ppm): δ 8.55 (d, *J* = 7.7 Hz, 1H), 7.75 (t, *J* = 7.7 Hz, 1H), 7.50 (d, *J* = 8.2 Hz, 1H), 7.43 (t, *J* = 7.6 Hz, 1H), 4.41 (d, *J* = 7.4 Hz, 2H), 2.31–2.22 (m, 1H), 1.51–1.47 (m, 2H), 1.28–1.18 (m, 30H), 0.86 (q, *J* = 7.1 Hz, 6H).

**^13^C NMR** (125 MHz, CDCl_3_, ppm): δ 150.60, 148.80, 147.12, 146.09, 145.22, 140.65, 135.75, 133.58, 132.02, 123.74, 121.56, 118.81, 110.24, 108.14, 46.30, 37.26, 32.05, 31.76, 30.08, 29.85, 29.75, 29.68, 29.63, 29.46, 26.36, 22.83, 14.27.

### 2.3. Synthesis of the Polymer PIQ

Quantities of 1,4-dibromo-2,3-difluoro-6-(2-octyldodecyl)-6*H*-indolo[2,3-*b*]quinoxaline (0.2 mmol, 0.1387 g), 2,5-bis(trimethylstannyl)thiophene (0.2 mmol, 0.0828 g), and toluene (6 mL) were added to a oven-dried Schlenk tube (100 mL) under argon atmosphere. The mixture was degassed with argon for 30 min. Next, Pd_2_(dba)_3_ (0.004 mmol, 0.0037 g) and P(*o*-tolyl)_3_ (0.016 mmol, 0.009 g) were added. After being degassed with argon for another 10 min, the tube was sealed. The tube was placed in a 110 °C oil bath. After 48 h, it was cooled down to room temperature. The reaction mixture was poured into stirring methanol to precipitate the crude product. The precipitate was collected by filtration and was further purified by sequential Soxhlet extractions with methanol and petroleum ether. The residue after Soxhlet extractions was then extracted with chloroform. The chloroform solution was re-precipitated with methanol. The resulting solid was collected and then dried to obtain the title polymer (0.0989 g, 80.3%) as a purple-black solid.

**^1^H NMR** (600 MHz, CDCl_3_, ppm): δ 8.43(br, 2H), 7.55–7.08(br, 4H), 4.27 (br, 2H), 2.02–0.88 (br, 38H).

**GPC** (THF): *M*_n_ = 7.1 kDa, *Ð* = 1.98.

***T*_d_** (5% loss) = 464 °C.

### 2.4. Device Fabrication and Characterization

The OPV device structure was set to ITO/PEDOT:PSS/**PIQ**:Y6/PDINN/Ag. The ITO glass substrates were ultrasonicated in deionized water with various reagents (acetone and 1,2-propanol), and dried in ambient atmosphere for 15 h. The dried glass substrates were treated with UV ozone for 20 min, and the PEDOT:PSS layers were spin-coated onto substrates at 7000 rpm for 60 s. The PEDOT:PSS layers had a thickness of 30 nm. Next, the film underwent an annealing process in the air at 150 °C for 15 min. The substrates were transferred into an Ar-filled glove box to spin-coat the active layers. The active layer materials **PIQ** and Y6 were dissolved in chloroform with a 1:1 weight ratio at a total concentration of 16.8 mg/mL. The solution of the **PIQ** and Y6 was subsequently spin-coated onto the hole transport layer (PEDOT: PSS), and the spin speed was 2000 rpm for 50s, to form ca. 80 nm uniform active layers. After that, the active layer needed to anneal for 12 min at 100 °C in the vacuum glove box. Finally, a thin PDINN layer (ca. 1 nm) and Ag (ca. 120 nm) were evaporated in a high vacuum chamber (ca. 4 × 10^−6^ torr). After this step, the device can be used for corresponding characterizations. Under AM1.5 solar illumination, *J*-*V* curves were measured by PV Test Solutions solar simulator. The external quantum efficiency (EQE) of the solar cells was tested using Zolix SolarCellScan 100.

## 3. Results and Discussion

**PIQ** can be synthesized with low cost via a three-step reaction from cheap raw materials. Two synthetic routes were explored to obtain monomer **3** with indoline-2,3-dione as the cheap raw material. *N*-alkylation reaction between 1-(2-octyldodecyl)indoline-2,3-dione and 9-(iodomethyl)nonadecane was used to synthesize compound **1** in a high yield of 97.4%. The acetic acid-catalyzed condensation reaction between compound **1** and 3,6-dibromo-4,5- difluorobenzene-1,2-diamine was conducted to synthesize monomer **3** in a low yield of 28.9%. Thus, monomer **3** was obtained with a low overall yield of 28% through this synthetic route. An improved route is to conduct the acetic acid-catalyzed condensation reaction followed by the N-alkylation reaction, as monomer **3** could be obtained with a reasonable overall yield of 46%. The Stille cross-coupling polycondensation of 2,5-bis(trimethylstannyl) thiophene and monomer **3** was performed to gain the target polymer **PIQ** as a purple-black solid (80.3% yield). The number average molecular weight and polydispersity index for **PIQ** were 7.1 kDa and 1.98, respectively, and **PIQ** had good solubility in common organic solvents. We performed synthesis cost calculations of the polymer **PIQ** using the model developed by Li et al. [36], which can be used as a rough indication of synthetic complexity. The results were displayed in Supporting Information (see Table S2). The cost of **PIQ** synthesized via route 1 is approximately 414.3 ¥/g, whereas the cost of **PIQ** synthesized via route 2 is approximately 241.1 ¥/g. The latter is significantly lower than the former, indicating that route 2 is the preferable route. As shown in Table S3, the synthesis cost of **PIQ** is compared to those of some famous polymer donors (e.g., PTQ10, PBDB-T, and PM6) [37,38,39].

### 3.1. Optical Properties

**Figure 2:**
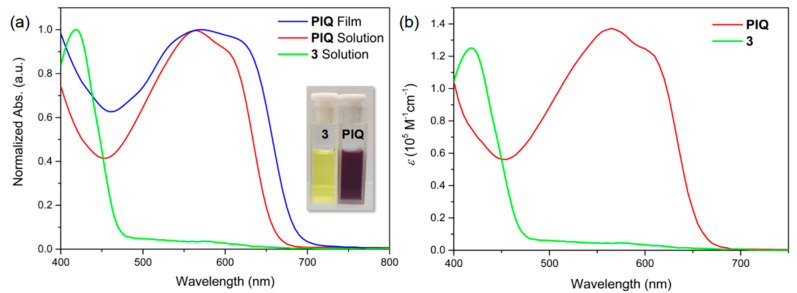
(**a**) Normalized UV-vis absorption spectra of monomer **3** and polymer **PIQ** in chlorobenzene solutions and **PIQ** as thin film (insert, photograph); (**b**) UV-vis absorption spectra of monomer **3** and **PIQ** in chlorobenzene solutions.

To study the optical properties of polymer **PIQ**, the UV-vis absorption spectra of monomer **3** and **PIQ** were tested. The photograph and absorption spectra of monomer **3** and polymer **PIQ** in dilute chlorobenzene solutions are shown in Figure 2a. **PIQ** solution exhibited absorption edge at 663 nm, which red-shifted over 188 nm relative to the monomer **3** solution. Introduction of the electron-donating thiophene to conjugated backbone can significantly enhance electronic delocalization along the chain axis via intramolecular charge transfer [40,41,42]. The monomer **3** solution has a strong absorption at 400–470 nm with a maximum absorption coefficient (ε) of 1.25×10^5^ M^−1^ cm^−1^, whereas the polymer **PIQ** solution has a much larger range of absorption, showing strong absorption in the 400–660 nm range with a slightly higher maximum absorption coefficient of 1.37 × 10^5^ M^−1^ cm^−1^ (Figure 2b). The absorption spectra of **PIQ** as a thin film is also displayed in Figure 2a. Compared to that of its solution, a distinct red shift by 22 nm was observed due to stronger aggregation in a solid state. The bandgap of **PIQ** as a thin film was estimated to be 1.80 eV, which could be comparable to that of PTQ10 and matched well with the typical low-bandgap acceptor of Y6 (see Figure S9 for its molecular structure) to show a complementary absorption [12,43]. The optical properties of **PIQ** are summarized in Table 1.

### 3.2. Electrochemical Properties

**Figure 3:**
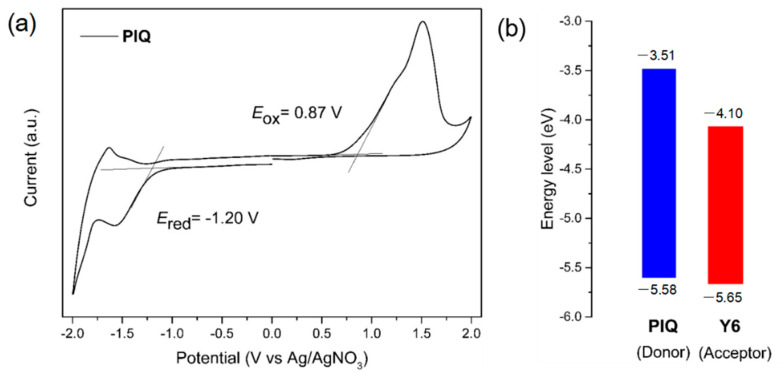
(**a**) Cyclic voltammetry curve of the **PIQ** thin film; (**b**) energy level diagram of the **PIQ** donor and Y6 acceptor.

The electronic energy levels of **PIQ** were measured by electrochemical cyclic voltammetry (Figure 3). The highest occupied molecular orbital (HOMO) and lowest unoccupied molecular (LUMO) levels of **PIQ** were estimated to be −5.58/−3.51 eV from the first onset oxidation and onset reduction potentials, respectively. The electrochemical properties of **PIQ** are also summarized in Table 1. The value of the electrochemical band gap for **PIQ** thin film was found to be 2.07 eV, which was larger than that of its optical band gap (1.8 eV). This may be due to the exciton binding energy for conjugated polymers [44].

### 3.3. Thermal Properties and X-ray Diffraction Characterization

The thermal stability of the **PIQ** polymer was tested by thermogravimetric analysis by taking approximately 6 mg of sample and placing it in an alumina ceramic crucible under nitrogen protection at a temperature increase rate of 20 °C/min up to 600 °C. The mass change of the sample at different temperatures was observed by heating. Organic polymer semiconductor materials can be considered to have good thermal stability when the temperature of 5% thermal weight loss is above 300 °C, which fully meets the requirements of optoelectronic device construction and testing. The temperature of 5% thermal weight loss of **PIQ** was 464 °C (see Figure S6), indicating that **PIQ** has good thermal stability.

To investigate the crystallinity of **PIQ** film, the X-ray diffraction (XRD) measurement was performed on a drop-cast film of **PIQ** (Figure S6). The sample showed distinct 100 peak at 5.17°, corresponding to a lamellar distance of 17.08 Å.

### 3.4. Photovoltaic Properties and Photoluminescence Characterization

**Figure 4:**
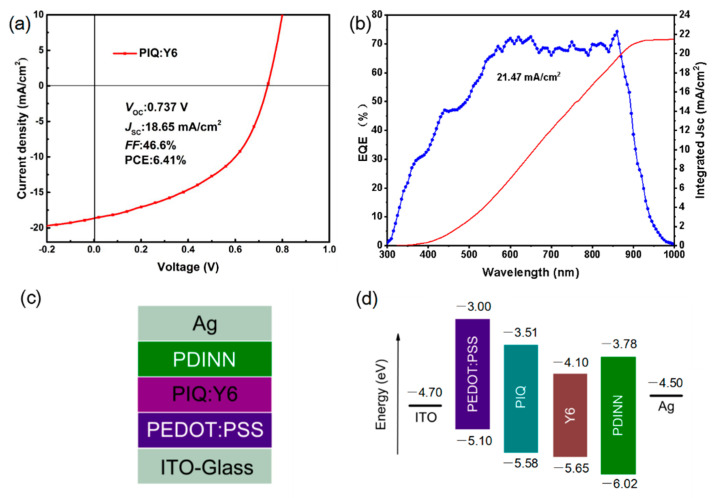
(**a**) *J-V* curve of the **PIQ**: Y6 blend-based polymer solar cells; (**b**) EQE spectrum of the PIQ: Y6 blend-based polymer solar cells; (**c**) schematic diagram of the device structure; (**d**) energy level diagram of the related materials.

**Figure 5:**
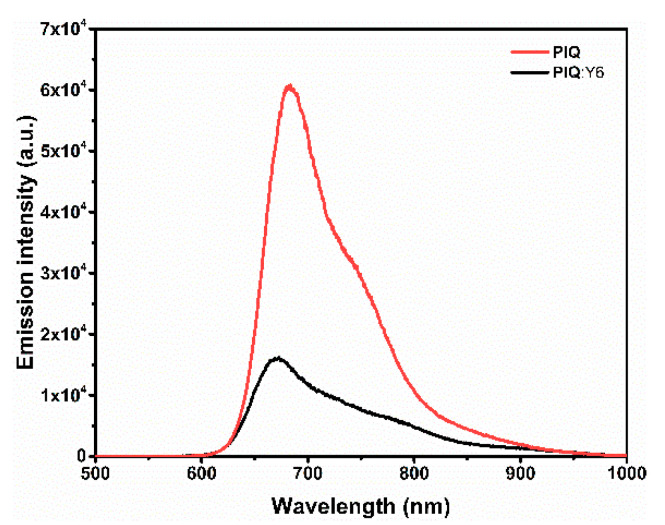
Thin-film photoluminescence spectra of **PIQ** and **PIQ**:Y6 blend.

To study the photovoltaic properties of PIQ, we fabricated BHJ polymer solar cells with a device structure of ITO/PEDOT:PSS/**PIQ**:Y6/PDINN/Ag (Figure 4c). The corresponding energy level diagram of the related materials is shown in Figure 4d. The polymer **PIQ** and Y6 were dissolved in chloroform. Devices with a donor/acceptor (D/A) ratio of 1:1 were fabricated. As illustrated in Figure 4a, a power conversion efficiency (PCE) of 6.41% was achieved with the fill factor (FF) of 46.6%, combined with the *J*_SC_ of 18.65 mA/cm^2^) and *V*_OC_ of 0.737 V. The *J*_SC_ value of polymer solar cells can be confirmed by the external quantum efficiency (EQE) measurement, and the result is shown in Figure 4b. Thin-film photoluminescence (PL) spectra of **PIQ**, **PIQ**:Y6 blend were measured (Figure 5). Blending PIQ with Y6 results in strong fluorescence quenching, indicating efficient photo-induced charge transfer [2,45] between **PIQ** and Y6 in blend.

## 4. Conclusions

In summary, a new polymer donor, **PIQ**, has been developed. **PIQ** can be easily gained via a simple three-step reaction from cheap raw materials with reasonable overall yield. **PIQ** has a medium bandgap of 1.80 eV, a broad and strong absorption feature in the wavelength range of 400~650 nm, and a low-lying HOMO energy level. The PSCs based on binary blend with **PIQ** as donor and Y6 as acceptor displayed a reasonable PCE of 6.41%. We believe that the tuning of physicochemical properties of the **PIQ** via optimization of conjugated backbones and side chains and its polymerization reaction may bring about further improvement in photovoltaic performance. We have developed the indoloquinoxaline-based polymer as the donor material for organic solar cells, and we also believe that indoloquinoxaline-based polymers can be promising low-cost and efficient polymer donor photovoltaic materials.

## Figures and Tables

**Table 1:** The optical and electrochemical properties of **PIQ**.

Polymer	*λ_max_^sol^* (nm)	*λ_max_^film^* (nm)	*λ_onset_^film^* (nm)	*E_g_^opt^*^1^ (eV)	*E_red_/E_LUMO_*^2^ (V/eV)	*E_ox_/E_HOMO_*^3^ (V/eV)
**PIQ**	564	570	687	1.80	−1.20/−3.51	0.87/−5.58

^1^ Calculated by the equation: $E_{g}^{opt}=\frac{1240}{\lambda_{onset}^{\mathrm{film}}}\;eV$; ^2^ $E_{LUMO}=-e\left(E_{red}+4.71\right)\;eV$; ^3^$\;E_{HOMO}=-e\left(E_{ox}+4.71\right)\;eV$.

## Data Availability

The data presented in this study are available on request from the corresponding author.

## Supplementary Materials

The following supporting information can be downloaded at: https://www.mdpi.com/article/10.3390/polym14081554/s1, Figures S1–S3: NMR spectrum; Figure S4: GPC test result of the polymer **PIQ**; Figure S5: TGA curve of the polymer **PIQ**; Figure S6: XRD pattern of the polymer **PIQ** thin film; Figure S7: Chemical structures of polymer donors involved in Table S1; Figure S8: Chemical structures of PBDB-T and PBDB-T-2F; Figure S9: Chemical structures of Y6; Table S1: Survey of polymer solar cells based on some representative low-cost and efficient donor polymers materials; Table S2: Survey of calculated chemical synthesis costs for **PIQ**; Table S3: Comparison of the synthetic steps and synthesis costs for polymer donor materials.
