# Supplementary material for: Synthesis of a Low-Cost Thiophene-Indoloquinoxaline Polymer Donor and Its Application to Polymer Solar Cells
Source: Polymers (Basel). 2022 Apr 11;14(8):1554. doi: 10.3390/polym14081554 (PMC9030569; doi:10.3390/polym14081554)
Supplement: Supplementary file 1 [file polymers-14-01554-s001.zip › polymers-1656395-supplementary.pdf]

Supplementary Materials: Synthesis of a Low-Cost Thiophene-Indoloquinoxaline Polymer Donor and Its Application to Polymer Solar Cells

Yiping Guo, Zeyang Li, Mengzhen Sha, Ping Deng, Xinyu Lin , Jun Li, Liang Zhang, Hang Yin and Hongbing Zhan

Materials and instruments

^{1}H NMR and ^{13}C NMR were recorded on Bruker AVIII 400 MHz or 600 MHz nuclear magnetic resonance instrument in deuterated chloroform ($CDCl_3$) with tetramethylsilane as the internal standard. UV-Vis spectra were measured by Perkinelmer LAMBDA 950 UV-Vis spectrophotometer. Photoluminescence (PL) spectra were taken on Horiba Jobin yvon FluoroMax-4 spectrometer. Cyclic voltammetry (CV) was tested on the Ingsens IGS1200 electrochemical analyzer, which had a three-electrode cell and was carried out in the deoxidation anhydrous acetonitrile solution of tetra-n-butylammonium-hexafluorophosphate (0.1 M) under nitrogen environment. Platinum disk electrode, platinum wire electrode and Ag/$AgNO_3$ electrode were used as a working electrode, a counter electrode and a reference electrode, respectively. The sample films for electrochemical measurements were coated on the surface of platinum electrode. The CV curves were calibrated using the ferrocene/ferrocenium (Fc/Fc^+) redox couple as an external standard, which was measured under same condition before and after the measurement of samples. The energy level of Fc/Fc^+ was assumed at –4.8 eV to vacuum. The half-wave potential of Fc/Fc+ was found to 0.09 V, related to the Ag/Ag^+ reference electrode. The HOMO energy levels of the polymer were calculated using the equation: E_{HOMO}= –e(4.71+E_{ox}) eV, where E_{ox} is the onset oxidation potential relative to the Ag/Ag^+ reference electrode. The LUMO energy levels of the polymer were calculated using the equation: E_{LUMO}= –e(4.71+E_{red}) eV, where E_{red} is the onset reduction potential relative to the Ag/Ag^+ reference electrode. Thermo-gravimetric analysis (TGA) was tested on NETZSCH STA449-F5 at a heating rate of 20 ^{o}C/min under nitrogen atmosphere. X-ray diffraction (XRD) data was taken on a Rigaku MiniFlex 600 diffractometer with copper K_α radiation.

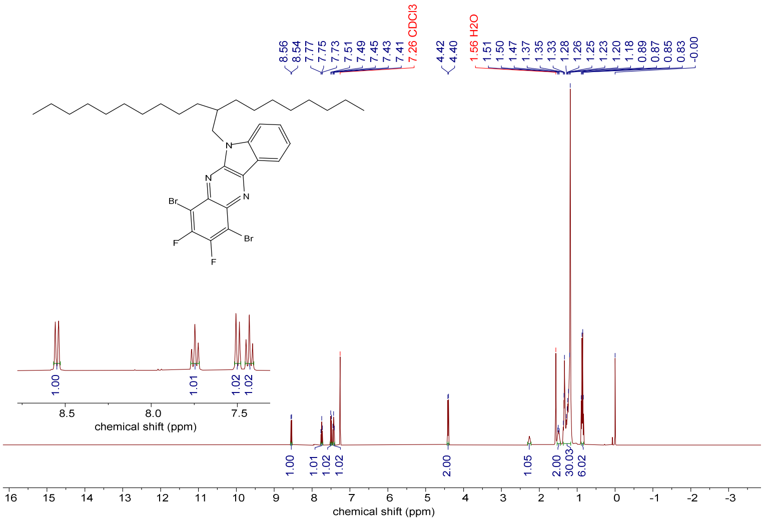

Figure S1. ^{1}H NMR spectrum of 1,4-dibromo-2,3-difluoro-6-(2-octyldodecyl)-6H-indolo[2,3-*b*]quinoxaline (in $CDCl_3$).

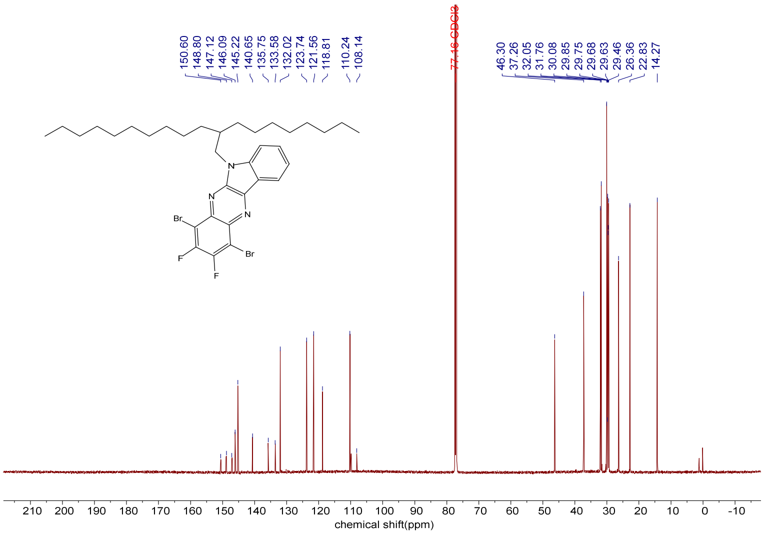

Figure S2. ^{13}C NMR spectrum of 1,4-dibromo-2,3-difluoro-6-(2-octyldodecyl)-6H-indolo[2,3-*b*]quinoxaline (in $CDCl_3$).

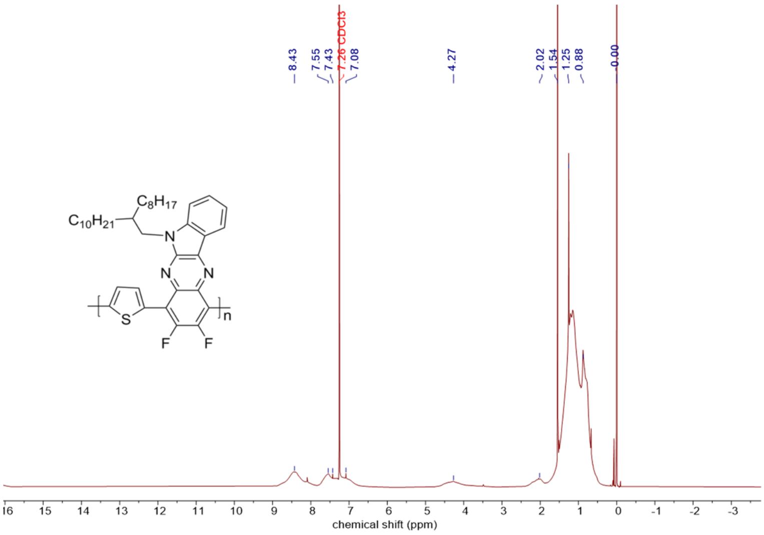

Figure S3. ^{1}H NMR spectrum of the polymer **PIQ** (in $CDCl_3$).

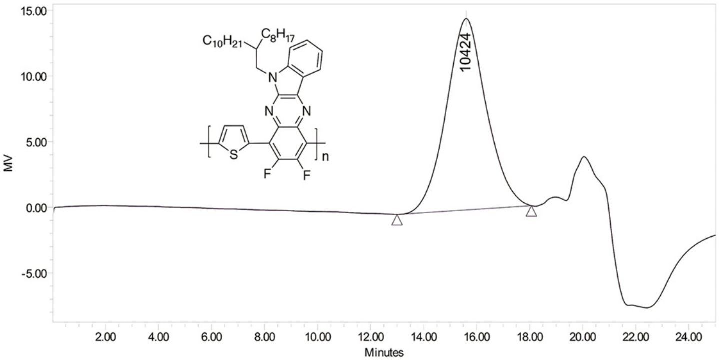

Broad Unknown Modified Universal Peak Table

	Distribution Name	Mv (Daltons)	K (dl/g)	alpha	Intrinsic Viscosity (dl/g)	Mn (Daltons)	Mw (Daltons)	MP (Daltons)	Mz (Daltons)	Mz+1 (Daltons)	Polydispersity	Mz/Mw
1						7108	14082	10424	26051	42611	1.981084	1.849855

Figure S4. GPC test result of the polymer **PIQ** (THF as eluent, 40 °C).

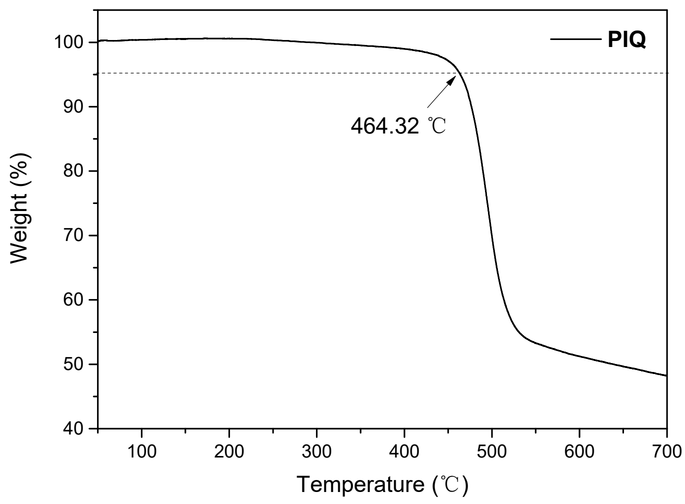

Figure S5. TGA curve of the polymer **PIQ**. (Heating rate: 20 °C/min; N_2 atmosphere).

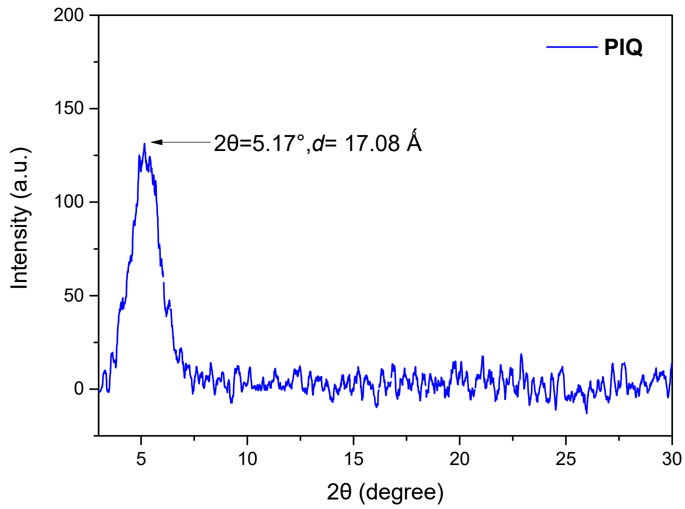

Figure S6. XRD pattern of the polymer **PIQ** thin film.

Table S1. Survey of polymer solar cells based on some representative low-cost and efficient donor polymers materials.

Donors	Acceptors	Anode	Hole Transport Layer	Electron Transport Layer	Cathode	PCE (%)	Reference
PTQ10	IDIC	Ag	MoO_3	ZnO	ITO	12.70	[12]
PTQ7	Y6	ITO	PEDOT:PSS	PDINO	Al	5.75	[16]
PTQ8	Y6	ITO	PEDOT:PSS	PDINO	Al	0.90	[16]
PTQ9	Y6	ITO	PEDOT:PSS	PDINO	Al	10.50	[16]
PTQ10	Y6	Ag	MoO_3	ZnO	ITO	15.03	[17]
P3HT	Y6	ITO	PEDOT:PSS	Ca	Al	2.41	[18]
PTOBT-Z	ITIC	Ag	MoO_3	ZnO	ITO	9.04	[19]
PTOBT-Z/E	ITIC	Ag	MoO_3	ZnO	ITO	6.86	[19]
PTVT-T	IT-4F	ITO	PEDOT:PSS	PFN-Br	Al	11.28	[20]
PTVT-T	eC9	ITO	PEDOT:PSS	PFN-Br	Al	16.20	[20]
P4T2F-HD	Y6-BO	Ag	MoO_3	ZnO	ITO	13.65	[21]
P301	Y5	ITO	PEDOT:PSS	PFN-Br	Al	2.33	[22]
P302	Y5	ITO	PEDOT:PSS	PFN-Br	Al	9.65	[22]

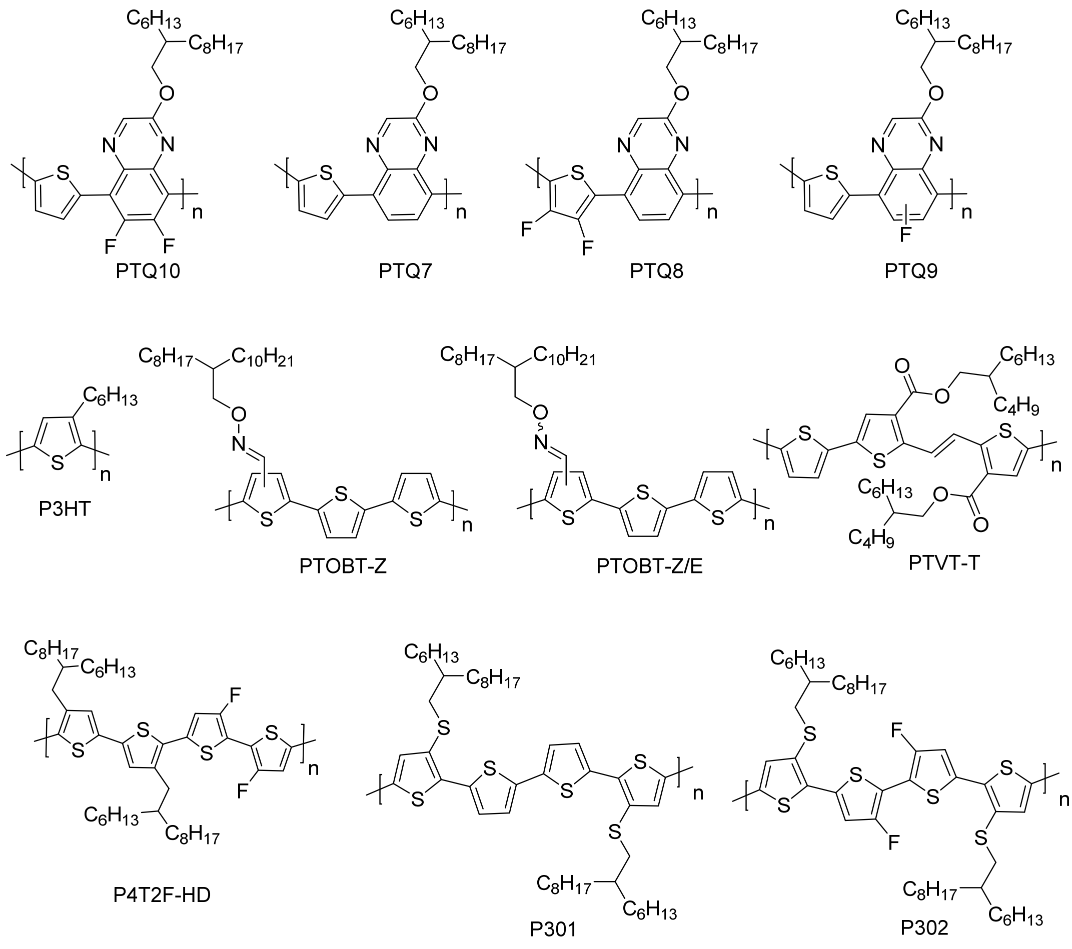

Figure S7. Chemical structures of polymer donors involved in **Table S1**.

Table S2. Survey of calculated chemical synthesis costs for **PIQ** (100 g) [1].

Path		Material	Quantity consumed	Cost (¥)	Total (¥)
Route 1	Reagents	Indoline-2,3-dione	102.7 g	91.4	41427.5
		9-(Iodomethyl)nonadecane	427.9 g	556.3	
		3,6-Dibromo-4,5-difluorobenzene-1,2-diamine	263.2 g	30268	
		thiophene	18 g	5	
		n-BuLi	0.17 L	170	
		trimethyltin chloride	0.42 L	4410	
		K_2CO_3	193.1 g	9.6	
		$Pd_2(dba)_3$	3.7 g	425.5	
		$P(o\text{-tolyl})_3$	9.1 g	92.8	
	Solvent	DMF	2.9 L	130.5	
		CH_3COOH	4.5 L	94.5	
		THF	0.4 L	30	
		Toluene	6.1 L	225.7	
	Purification	$MgSO_4$	320 g	28.7	
		Eluent and extraction solvent	216 L	2592	
		SiO_2	53.4 kg	1335	
		Methanol	15 L	255	
		Petroleum ether	15 L	120	
		Chloroform	15 L	516	
	other	Calcium hydride	60 g	26	
		molecular sieves	0.6 kg	45.5	
Route 2	Reagents	Indoline-2,3-dione	78.1 g	69.5	24114.9
		9-(Iodomethyl)nonadecane	213.5 g	277.6	
		3,6-Dibromo-4,5-difluorobenzene-1,2-diamine	133.5 g	15352.5	
		thiophene	18 g	5	

	n-BuLi	0.17 L	170
	trimethyltin chloride	0.42 L	4410
	K_2CO_3	93.7 g	4.7
	$Pd_2(dba)_3$	3.7 g	425.5
	$P(o\text{-tolyl})_3$	9.1 g	92.8
Solvent	DMF	1.5 L	67.5
	CH_3COOH	1.4 L	29.4
	THF	0.4 L	30
	Toluene	6.1 L	225.7
Purification	$MgSO_4$	320 g	28.7
	Eluent and extraction solvent	108 L	1296
	SiO_2	26.7 kg	667.5
	Methanol	15 L	255
	Petroleum ether	15 L	120
	Chloroform	15 L	516
other	Calcium hydride	60 g	26
	molecular sieves	0.6 kg	45.5

[1] The chemical synthesis cost was calculated according to the model reported in reference 36.

Table S3. Comparison of the synthetic steps and synthesis costs for donor materials.

Compound	Total Step	C_g (¥ per g) [1]	Reference
**PIQ**	4	241.1	This work
PTQ10	3	259.3	[12,36]
P3HT	2	70.1	[36,37]
PBDB-T [2]	10	367.2	[36,38]
PM6 [2]	11	778.7	[36,39]

[1] C_g cost-per-gram, and unit cost converted from the calculation; [2] Donors commonly used in polymer solar cells, their molecular structures are shown in Fig. S8.

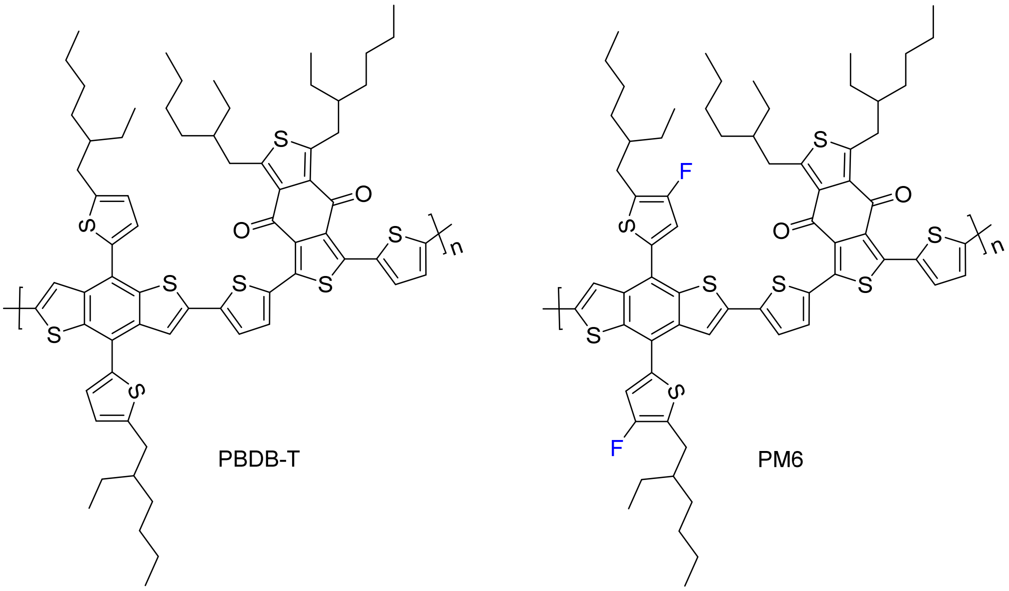

Figure S8. Chemical structures of PBDB-T and PM6.

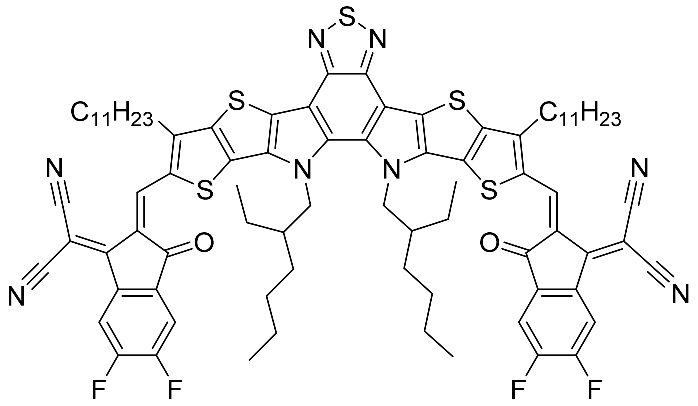

Figure S9. Chemical structure of Y6.
